# Unique Cytopathological Findings of a Mucinous Myoepithelioma Showing a Mucous Globule and a "Sieve-Like" Structure

**DOI:** 10.7759/cureus.67697

**Published:** 2024-08-24

**Authors:** Takeru Oyama, Akihiro Shioya, Hiroko Ikeda, Daichi Maeda, Sohsuke Yamada

**Affiliations:** 1 Department of Pathology and Laboratory Medicine, School of Medicine, Kanazawa Medical University, Uchinada, JPN; 2 Department of Diagnostic Pathology, Kanazawa University Hospital, Kanazawa, JPN; 3 Department of Molecular and Cellular Pathology, Kanazawa University, Graduate School of Medical Science, Kanazawa, JPN

**Keywords:** subtypes and variants, cribriform, sieve-like, mucous globule, mucinous myoepithelioma

## Abstract

Myoepitheliomas are rare salivary gland-type tumors. The tumors are divided into four histological subtypes (spindle cell, plasmacytoid, epithelioid, and clear cell) and two variants (reticular and mucinous). A myoepithelioma of the mucinous variant, also referred to as mucinous or secretory myoepithelioma, is a novel variant of myoepithelioma characterized by the presence of extracellular mucin. To date, only six benign mucinous myoepitheliomas have been reported. We herein report an 84-year-old man with a four-month history of swelling in the left bucca. Computed tomography revealed a well-demarcated elliptical mass measuring 30 mm in diameter. Fine-needle aspiration (FNA) smears showed an acellular spherical matrix surrounded by basaloid cells with scant cytoplasm resembling mucous globules, in addition to clusters of spindle cells. The mass was initially diagnosed as a pleomorphic adenoma based on the presence of a mucous globule-like structure and cytological variation. The surgically resected tumor showed two different histological components: one was composed of cells arranged in thin cords with a mucoid stroma showing a “sieve-like” structure and the other component was spindle cells. Alcian blue staining confirmed extracellular mucin in both tumor components. The tumor was suspected of being a mucinous myoepithelioma. We encountered a case of a mucinous myoepithelioma with two unique features, namely its cytological features of mucous globules composed of monotonous basaloid cells in the FNA smear and its histological feature of a “sieve-like” structure. The presence of mucous globules in FNA smears might require the inclusion of the mucinous myoepithelioma in the differential diagnosis.

## Introduction

A myoepithelioma is an uncommon tumor, comprising 1.5% of all salivary gland tumors [1,2]. The age at presentation is 9-85 years old, with an average age of 44 years old and no sex predilection [3]. The tumor occurs mostly in the major salivary gland, followed by the soft and hard palates [4]. Grossly, the tumor shows a well-demarcated, capsulated lesion, and its cut surface shows a solid mass, tan to yellow in color. Histologically, the tumor shows four subtypes (spindle cell [5], plasmacytoid [6], epithelioid [7], clear cell [8]) and two variants (reticular [9] and mucinous [10]). The spindle cell subtype is composed of spindle cells arranged in an interlacing fascicular pattern. The plasmacytoid subtype is composed of plasmacytoid cells with various amounts of hyaline stroma. The epithelioid subtype is composed of cells with abundant polygonal eosinophilic cytoplasm arranged in cords or nests. The clear cell subtype is composed of cells with glycogen accumulation in the cytoplasm. The reticular variant shows an irregular multilayered pseudoglandular structure accompanied by focal compact clusters of tumor cells, and mucinous myoepithelioma is defined by extracellular mucin.

A mucinous myoepithelioma, also known as a secretory myoepithelioma, is a novel variant of myoepithelioma coined by Gnepp characterized by the presence of extracellular mucin instead of hyaline material [2].

In the present case, the tumor histologically included a component of cells arranged in a “sieve-like” structure and cytologically showed mucous globules composed of single-layered uniform basaloid cells. Mucous globules, a known characteristic of neoplasms harboring pseudoglandular structures, such as adenoid cystic carcinoma, have hitherto not been reported for mucinous myoepitheliomas.

## Case presentation

**Figure 1:**
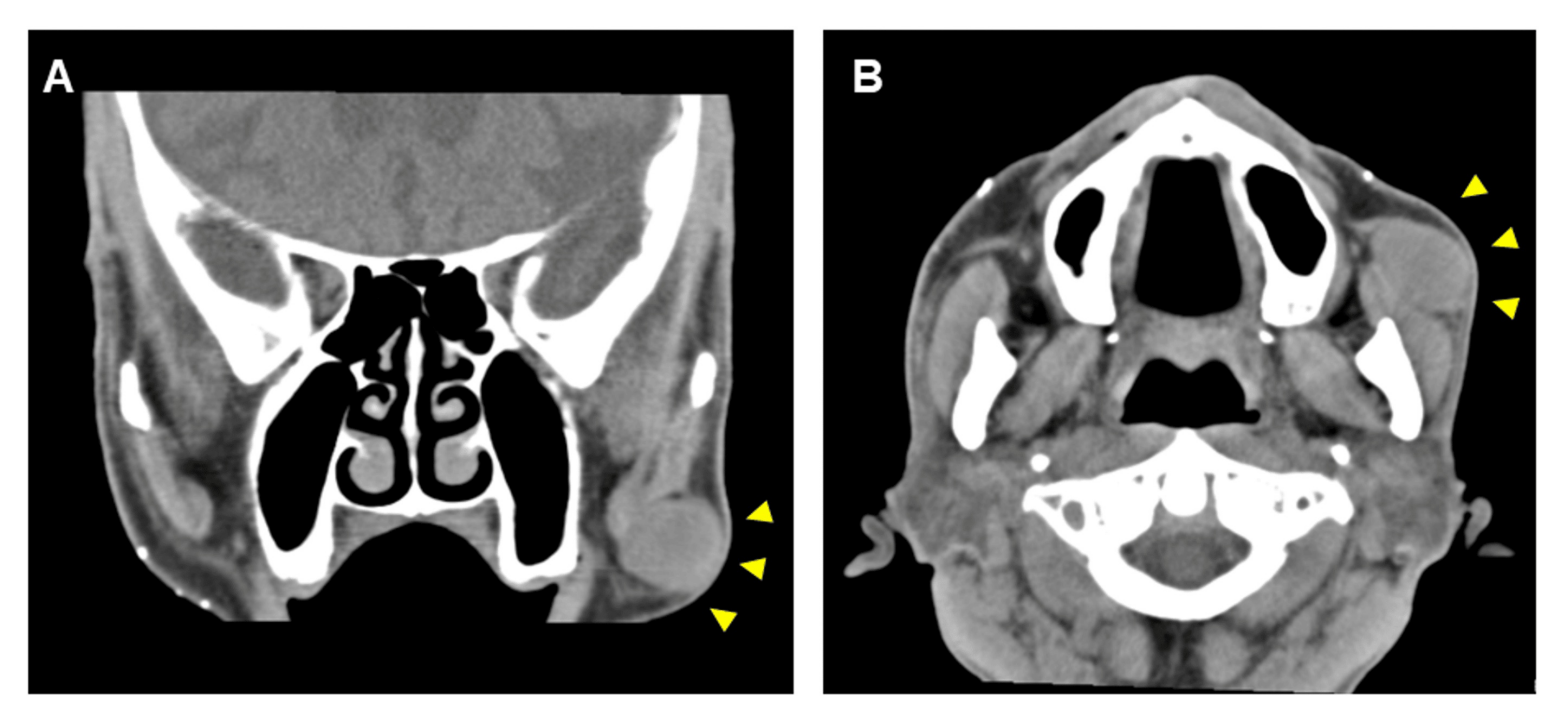
Computed tomography scan of the head and neck. A well-circumscribed iso-dense lesion approximately 30 mm in diameter was observed. A: Coronal section; B: Axial section. Yellow arrowheads indicate the lesion.

**Figure 2:**
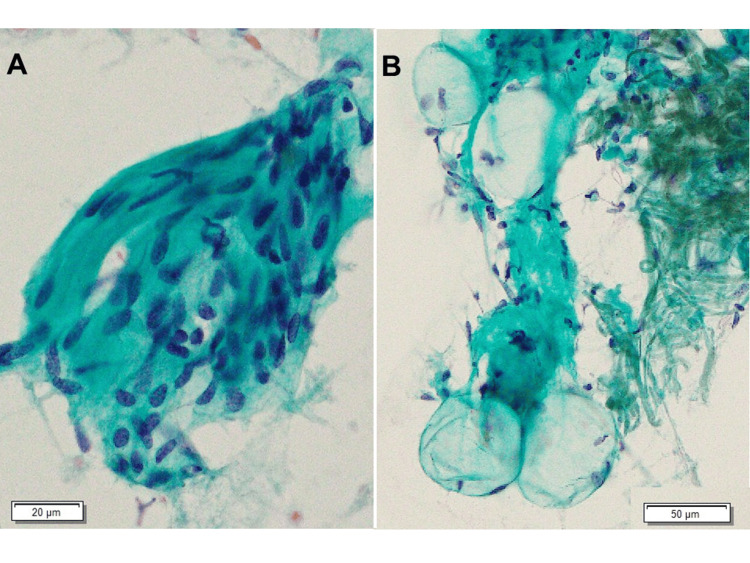
Fine-needle aspiration smears. The smears consisted predominantly of spindle cells (A) and mucous globules surrounded by single-layered uniform basaloid cells (B).

An 84-year-old man presented with swelling in his left bucca for four months. Computed tomography revealed a well-demarcated elliptical mass 30 mm in diameter on the left superficial lobe of the parotid gland (Figures 1A, 1B). Due to the possibility of salivary gland tumors, fine-needle aspiration (FNA) cytology was performed. The aspiration smears showed high cellularity and a relatively large cluster of spindle cells. Spindle cells were arranged in an interlacing fascicular pattern with uniform hyperchromatic spindle nuclei in the center of the fibrillary matrix (Figure 2A). A small number of acellular spherical matrices surrounded by basaloid cells resembling mucous globules was observed (Figure 2B). The cells showed uniform hyperchromatic nuclei with scant cytoplasm. The initial cytological diagnosis suggested pleomorphic adenoma because of the presence of mucous globule-like structures and various types of tumor cells.

**Figure 3:**
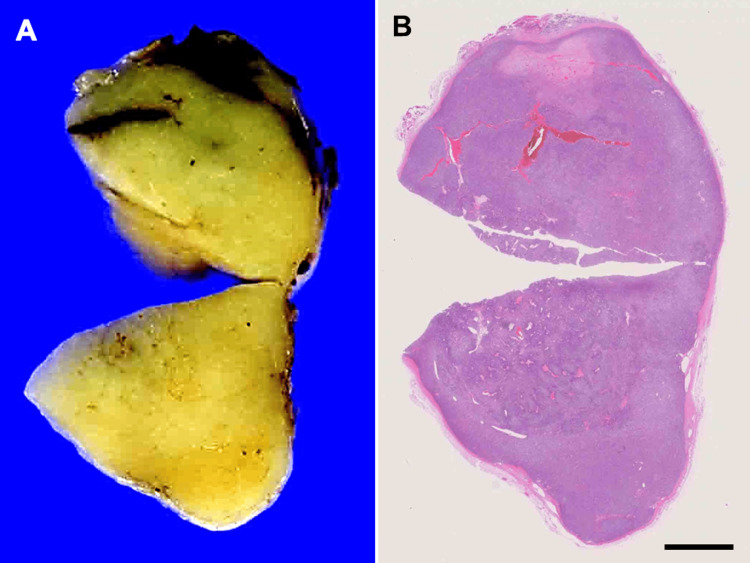
Cut surface (A) and loupe magnification (B) of the tumor. The cut surface shows heterogeneity in color, with a more bright-yellowish area in the center of the tumor. The tumor is surrounded by a fibrous capsule. Bar: 5mm

The patient underwent surgical resection of the mass to reduce the risk of malignant transformation of pleomorphic adenoma (1.5% in the first five years, 9.5% after 15 years) [11]. Following preservation of the left facial nerve, the mass was enucleated with a 5-mm surgical margin. A gross examination showed that the mass was an encapsulated, round, smooth, and elastic hard tumor, and the cut surface was yellowish white and uniform (Figure 3A).

**Figure 4:**
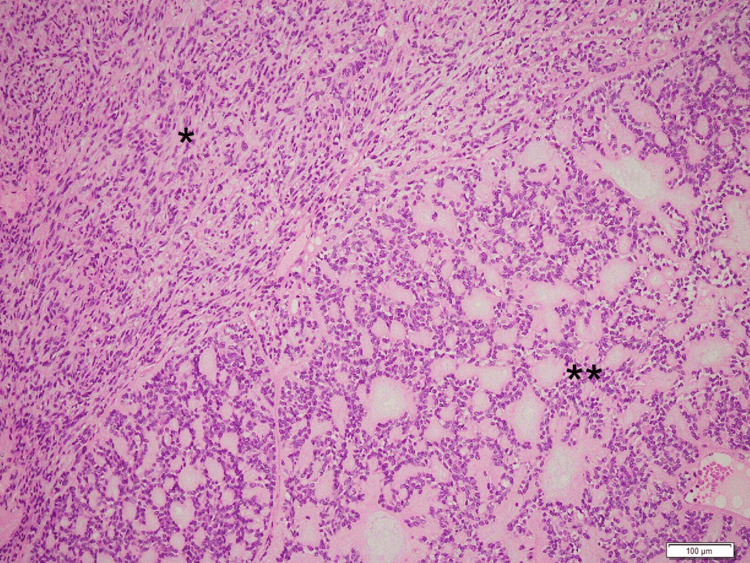
Hematoxylin and eosin-staining section of the tumor. The tumor is composed of two components: one composed of aggregation of spindle cells (*) and the other a “sieve-like” structure (**) surrounded by myxoid stroma.

**Figure 5:**
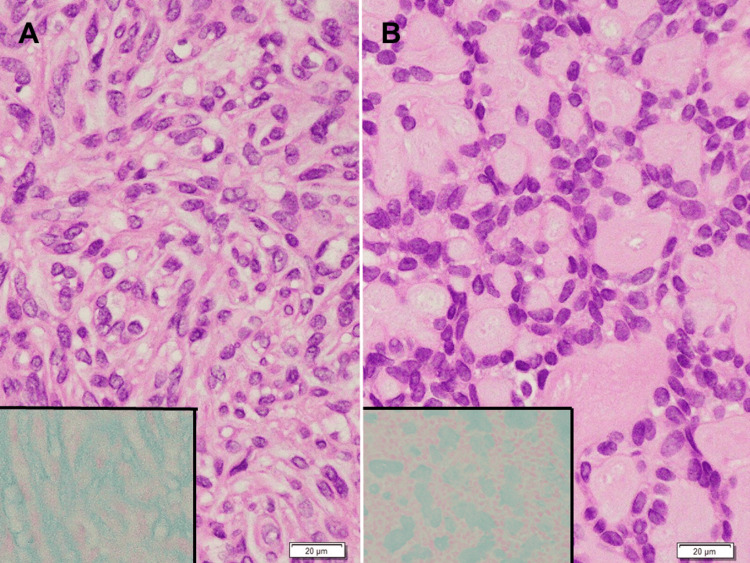
Hematoxylin and eosin-staining and alcian blue staining section of the tumor (high magnification). (A) Spindle cells arranged in an interlacing fascicular pattern with uniform hyperchromatic spindle nuclei in the center of a fibrillary matrix. (B) Interconnected cord of basaloid cells with round-to-oval nuclei, arranged in a “sieve-like” structure. Inserts: Alcian blue staining showed the existence of alcian blue-positive basophilic mucin in the extracellular spaces of both of the histological components.

A histological examination revealed that the tumor was encapsulated by a fibrous capsule, with no apparent invasion of the surrounding salivary gland observed (Figure 3B). The tumor was composed of two different components (Figure 4): one composed of spindle cells arranged in an interlacing fascicular pattern and showing uniform hyperchromatic spindle nuclei at the center of the fibrillary cytoplasm (Figure 5A) and the other composed of cells arranged in interconnected thin cords surrounded by a small, round space of mucoid stroma showing a “sieve-like” structure (Figure 5B). The cells had uniform hyperchromatic nuclei and inconspicuous nucleoli. Alcian blue staining showed extracellular mucin in both components (Figure 5A, 5B, inserts). These features suggested a benign mucinous myoepithelioma.

Immunostaining was performed for antibodies against cytokeratin (CKs) AE1/AE3, CK5/6, CK7, epithelial membrane antigen (EMA), p63, α-smooth muscle actin (SMA), muscle-specific actin (clone HHF-35), S-100 protein, glial fibrillary acidic protein (GFAP), and Ki-67 using VENTANA BenchMark ULTRA (Roche Diagnostics, Tokyo, Japan) according to standard protocols [12]. The tumor was positive for epithelial cell markers CK AE1/AE3, CK 5/6, CK7, myoepithelial cell markers p63 and α-smooth muscle actin, and markers of neoplastic myoepithelial cells, S-100 protein, and GFAP. All tumor cells were negative for EMA, a marker of the salivary duct. CKs (CK AE1/AE3, CK 5/6 and CK 7), p63 and S-100 protein showed positivity for almost all tumor cells; by contrast, α-SMA showed diffuse positivity for the component composed of spindle cells and focal positivity for the component showing a “sieve-like” structure. The Ki-67 labeling index was approximately 1%.

The results of immunostaining indicated myoepithelial differentiation and benignancy of the tumor cells, which were consistent with the diagnosis of mucinous myoepithelioma. Taken together, these findings suggest that the tumor in the present case harbored cytological and histological features as well as a pattern of immunohistochemical expression of benign mucinous myoepithelioma.

No recurrence was observed for 11 years after surgery.

## Discussion

Myoepitheliomas are uncommon salivary gland tumors composed of cells with myoepithelial differentiation. The tumor was located at one end of the pleomorphic adenoma spectrum. The majority of previous reports classified tumors with even a minority of duct structures into the tumor category. In contrast, several reports did not allow any ductal structures in a myoepithelioma, instead treating such tumors as myoepithelial-predominant pleomorphic adenomas [2]. Myoepitheliomas are composed mainly of spindle, plasmacytoid, and epithelioid cells with minor clear, oncocytic, and basaloid cells. The tumor cells are typically arranged in a solid, myxoid, reticular, or mixed pattern, and the stroma is either myxoid or hyalinized [13]. The tumor is divided into four histological subtypes (spindle cell, plasmacytoid, epithelioid, and clear cell) and two variants (reticular and mucinous) [9,10], and the four subtypes are divided by the tumor cell shape rather than the arrangement of tumor cells, whereas the two variants are categorized by cell arrangement or stromal properties.

Mucinous (secretory) myoepithelioma was coined in 2012 as a subtype of myoepithelioma that contains intracellular mucin. The tumor was considered a benign to low-grade malignancy [2]. Because of its infrequency, the same histological evaluation as other myoepitheliomas should be used for mucinous myoepithelioma. The majority of mucinous myoepitheliomas are composed of plasmacytoid cells with abundant eosinophilic to foamy grayish-blue cytoplasm and/or a cell resembling a signet ring cell, with mucin vacuoles and eccentric crescent-shaped nuclei [2]. The cells were arranged in nests and sheets, although only a small number of reported cases are available in the published literature.

Before 2013, 17 mucinous myoepitheliomas had been reported, of which 4 were classified as (benign) mucinous myoepitheliomas. Guo et al. in 2020 described a case of mucinous myoepithelioma composed of spindle and epithelioid cells with cytoplasmic mucin-filled vacuoles arranged in a solid and reticular pattern [1]. Val-Bernal et al. in 2022 described a case of a tumor composed of epithelioid cells arranged in a reticular pattern. Epithelioid cells showed no mucin vacuoles [13].

The tumor in the present case was initially classified as a benign myoepithelioma, fully encapsulated and composed of uniform spindle cells arranged in a solid pattern and basaloid cells arranged in a reticular-like pattern, without any ductal structures. The results of immunostaining were consistent with benign myoepithelioma: almost all tumors were positive for epithelial cell markers (CK AE1/AE3, CK 5/6, CK7), myoepithelial cell markers (p63), and markers of neoplastic myoepithelial cells (S-100 protein, and GFAP). The Ki-67 labeling index was <10% (approximately 1%). Furthermore, the tumor was classified as a mucinous myoepithelioma based on an examination of an intracellular mucoid material specimen, as shown by alcian blue staining.

The mucinous myoepithelioma observed in the present case exhibited unique histological findings. In the context of cell shape, the present tumor cells harbored scant cytoplasm or were presented as bare nuclei. The tumor cells showed no spindle, plasmacytoid, or clear cell shapes and did not harbor the epithelioid subtype, which is typically characterized by cells with abundant polygonal eosinophilic cytoplasm and centrally placed round to elliptical nuclei arranged in cords or nests [6]. In the context of the arrangement of tumor cells, the present tumor cells were arranged in thin cords, showing a “sieve-like” structure that did not fit the reticular variant. Regarding the reticular variant, Dardick et al. described four cases of reticular variants of myoepithelioma [10]. These tumors appeared to form irregular, multilayered pseudoglandular structures accompanied by focal compact clusters of tumor cells separated by myxoid and vascularized stroma. The tumors were composed of plump, relatively short spindle cells. Conversely, the “sieve-like” structure of the present case was composed of relatively regular, single-layered basaloid cells surrounding small round myxoid stroma (Figure 5B). In short, the “sieve-like” structure of the tumor suggests a potentially distinct morphological structure.

The mucinous myoepithelioma observed in the present case also exhibited unique cytological findings. The FNA smear of the tumor showed mucous globules in which mucous balls were surrounded by cells with uniform hyperchromatic nuclei and scant cytoplasm resembling basal cells. The mucous globules of the present case might mirror the histological pattern of “sieve-like” structures. In general, mucous globules show a pattern in which the globules of mucous balls are surrounded by tumor cells, which are characteristic of adenoid cystic carcinoma [14], pleomorphic adenoma [15], epithelial-myoepithelial carcinoma [16], and basal cell adenoma [17]. The globules of these tumors are surrounded by biphasic, myoepithelial, and glandular epithelial cells. In contrast, the mucous globules of the present tumor were surrounded by uniform, single-layered basaloid cells. 

## Conclusions

We herein described a case of a mucinous myoepithelioma, a novel variant of myoepithelioma, with two unique findings: namely, cytological findings of mucous globules surrounded by single-layered basaloid cells and histological findings of a “sieve-like” structure. The presence of mucous globules in initial FNA smears suggests that the mucinous myoepithelioma should therefore be included in the differential diagnosis of salivary gland tumors comprising malignancies, particularly adenoid cystic carcinoma and epithelial-myoepithelial carcinoma.
